# Primary Pulmonary Paraganglioma: A Rare Clinical Entity

**DOI:** 10.7759/cureus.102198

**Published:** 2026-01-24

**Authors:** Mahesh Murali, Antonious Selvam, Sibi Sam, Sebin J Thampan

**Affiliations:** 1 Pulmonology, Pondicherry Institute of Medical Sciences, Puducherry, IND; 2 Pulmonary Medicine, Pondicherry Institute of Medical Sciences, Puducherry, IND; 3 Pulmonary Medicine, Holy Family Hospital, Thodupuzha, IND

**Keywords:** immunohistochemical markers, immunohistochemistry, lung mass, neuroendocrine tumor, paraganglioma, primary pulmonary paraganglioma, sporadic paraganglioma

## Abstract

Paragangliomas are rare neuroendocrine tumors arising from extra-adrenal paraganglionic cells, with an estimated annual incidence of 2-8 cases per million. Primary pulmonary paragangliomas are exceptionally uncommon and are frequently mistaken for more prevalent pulmonary diseases, leading to diagnostic delays and management challenges. We report the case of a 60-year-old woman who presented with diffuse, non-radiating left-sided chest pain, progressive dyspnea transitioning from Modified Medical Research Council (mMRC) grades 0 to I, and unintentional weight loss over a two-year period. Laboratory investigations were unremarkable. Imaging demonstrated a well-defined mass in the left upper zone on chest radiograph, and contrast-enhanced computed tomography (CT) revealed a smoothly marginated, heterogeneously enhancing soft tissue lesion in the left upper lobe. Bronchoscopic biopsy initially suggested a clear cell neoplasm; however, immunohistochemical analysis demonstrated findings consistent with paraganglioma. The absence of clinical features suggestive of hereditary syndromes supported the diagnosis of a sporadic, non-functional pulmonary paraganglioma. This case underscores the importance of considering paraganglioma in the differential diagnosis of pulmonary masses and highlights the critical diagnostic role of immunohistochemistry in distinguishing this rare entity from morphologically similar tumors.

## Introduction

Paraganglioma is a rare disease that affects two to eight people per million per year [1]. Paragangliomas are neuroendocrine tumors that arise from the extra-adrenal paraganglionic cells. Pulmonary paragangliomas are difficult to differentiate from bronchial carcinomas and metastatic tumors. Tumors arising from chromaffin cells are located in about 90% of cases within the adrenal gland as pheochromocytomas, while the remaining extra-adrenal tumors, known as paragangliomas, originate from non-epithelial chromaffin cells [2]. Due to its rarity, primary pulmonary paraganglioma is often misdiagnosed with other more common pulmonary conditions, complicating clinical management [3].

## Case presentation

**Figure 1:**
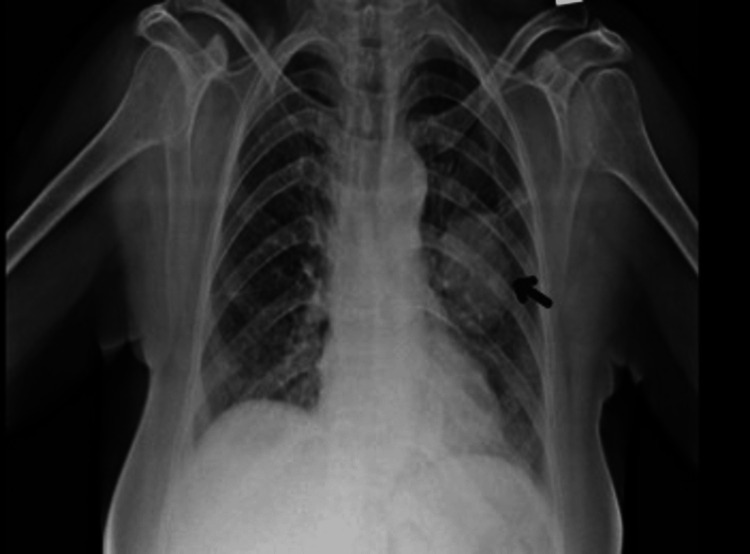
Chest radiograph showing a well-defined mass-like lesion in the left upper zone

**Figure 2:**
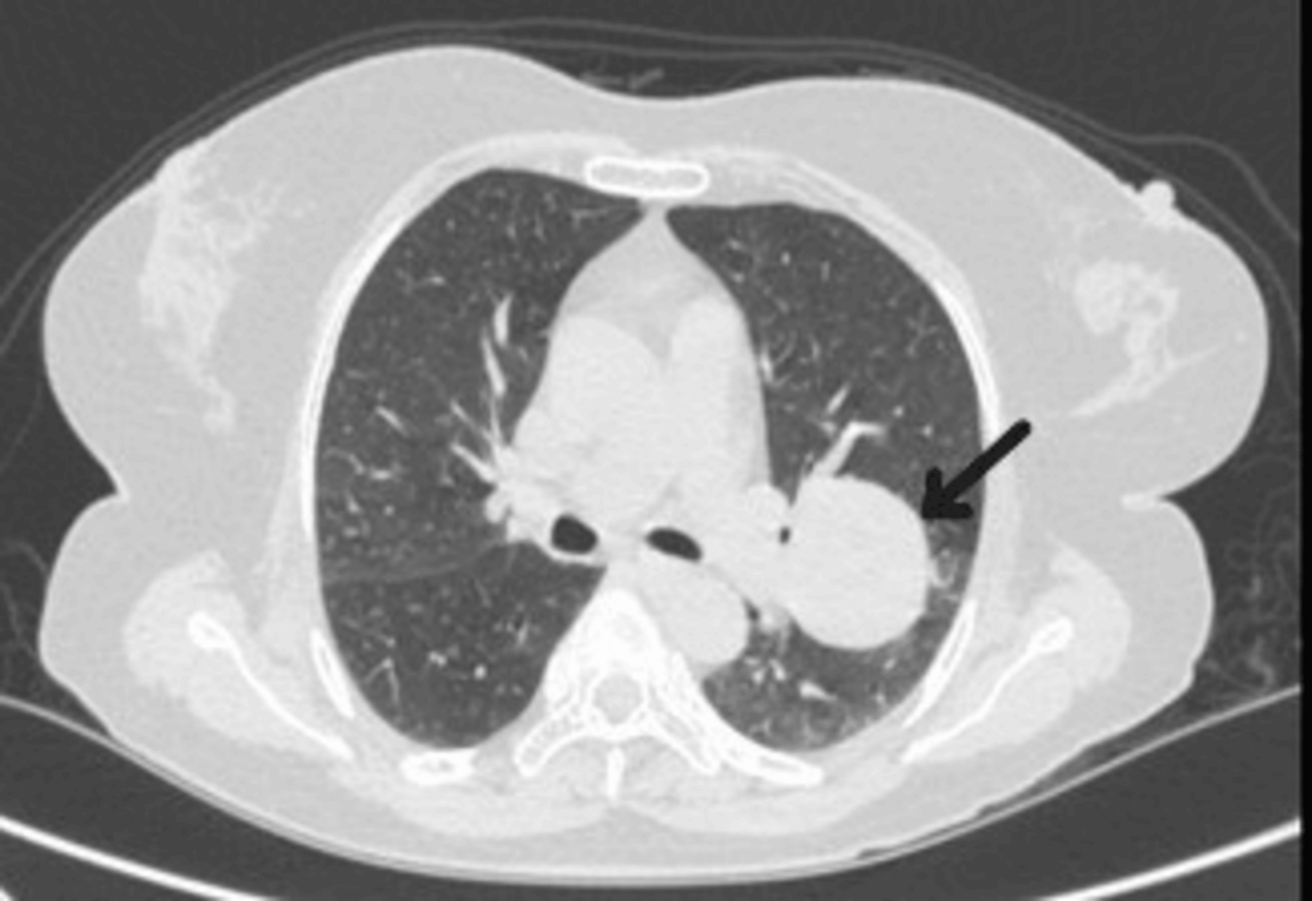
Contrast-enhanced computed tomography of the thorax axial section (lung window) showing a well-defined smoothly marginated heterogeneously enhancing soft tissue density lesion in the left upper lobe

**Figure 3:**
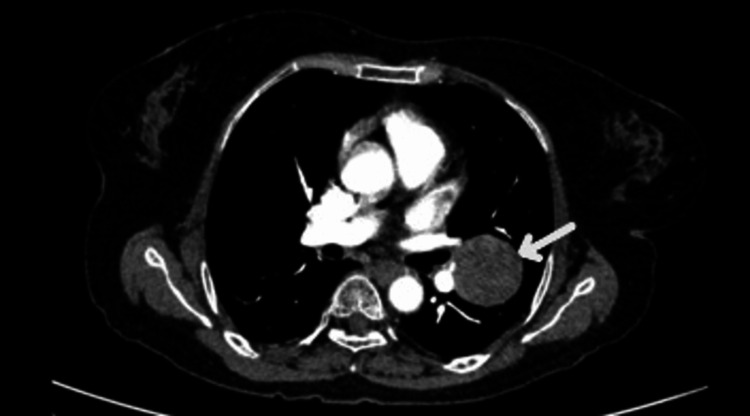
Contrast-enhanced computed tomography of the thorax axial section (mediastinal window) showing a well-defined smoothly marginated heterogeneously enhancing soft tissue density lesion in the left upper lobe

**Figure 4:**
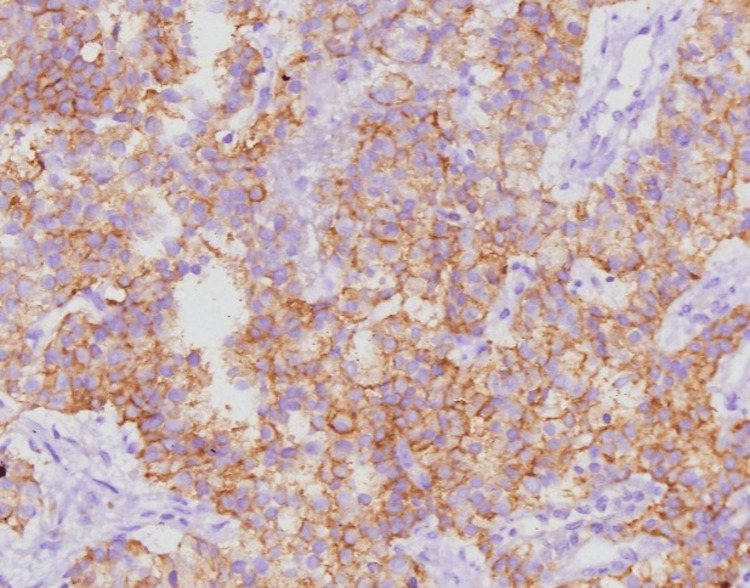
Immunohistochemistry showing positive CD56 staining

**Figure 5:**
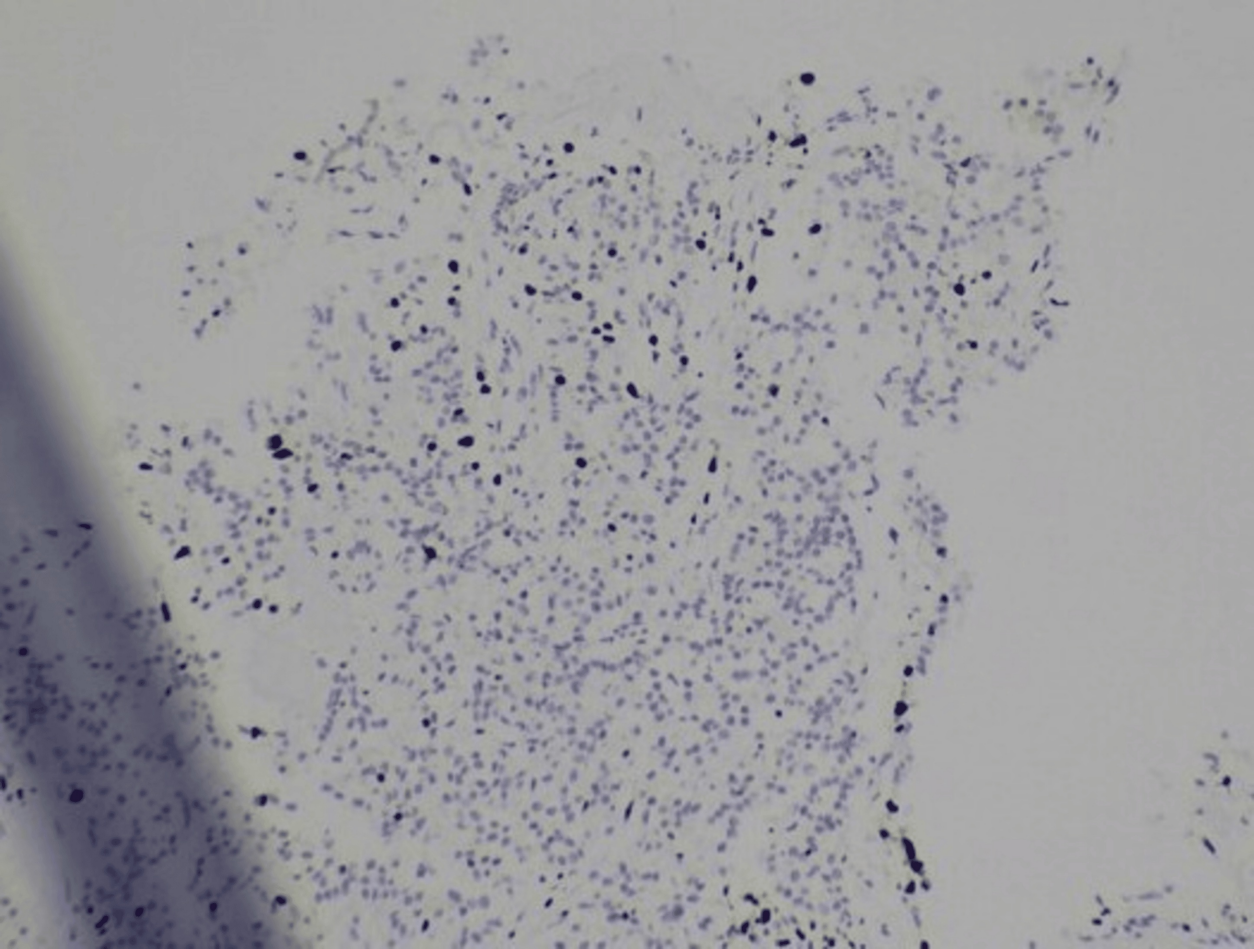
Immunohistochemistry showing positive Ki-67 staining

**Figure 6:**
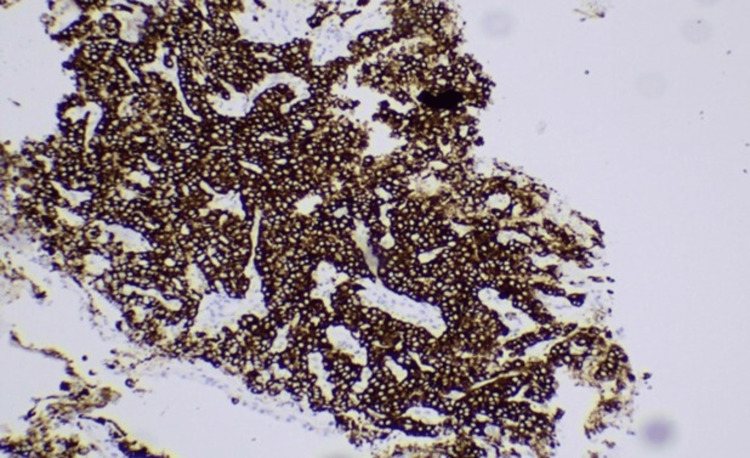
Immunohistochemistry showing positive synaptophysin staining

**Figure 7:**
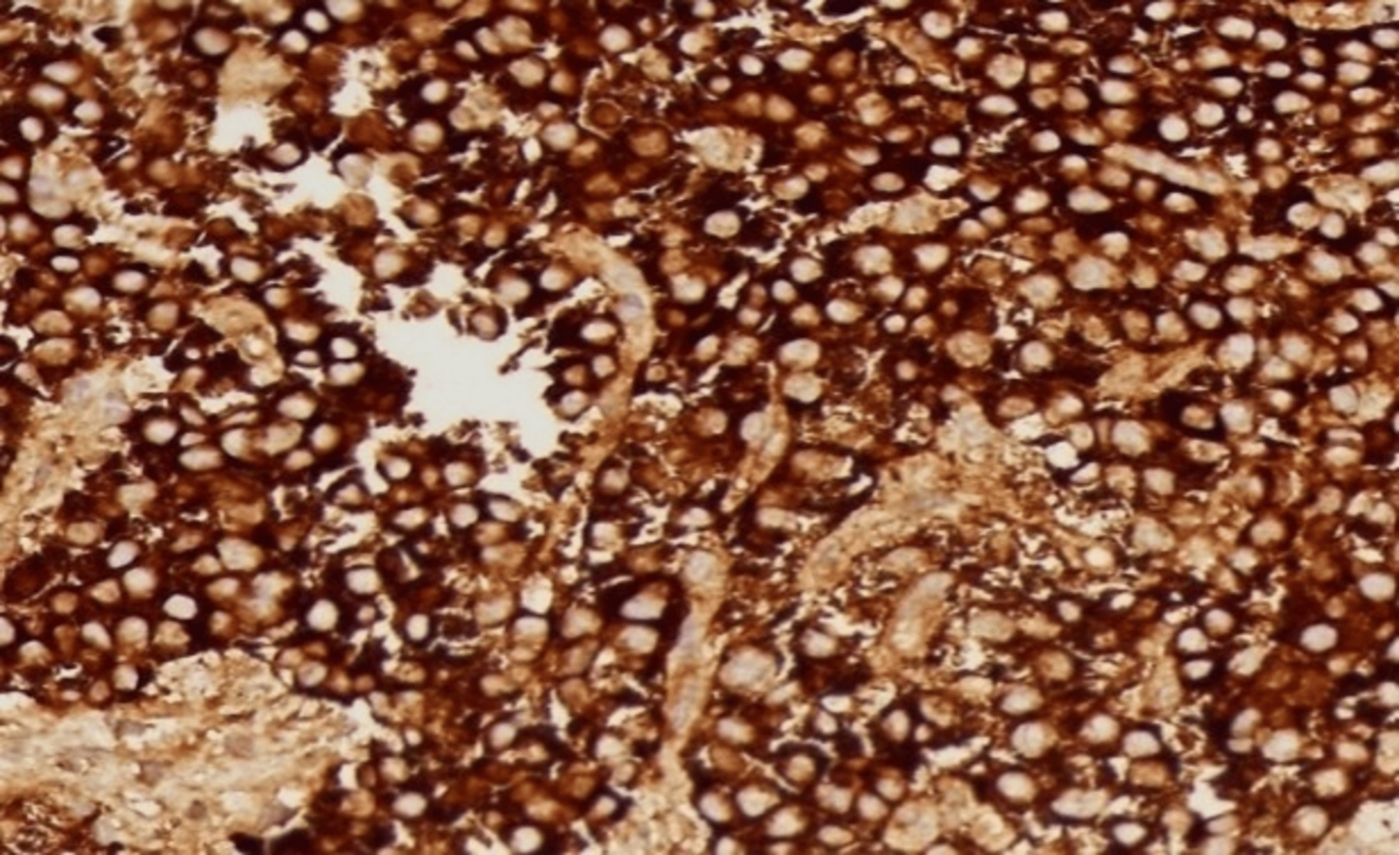
Immunohistochemistry showing positive chromogranin staining

A 60-year-old woman presented with complaints of diffuse non-radiating left-sided chest pain, progressive shortness of breath from Modified Medical Research Council (mMRC) grades 0 to I, and unintentional weight loss for two years. Her past medical history was unremarkable. There were no features suggestive of autoimmunity. There was no history of malignancy in the family. On receiving, her vitals were stable, and respiratory examination showed bronchial breath sounds over the infraclavicular and mammary areas with increased vocal resonance. Other systemic examinations were unremarkable with no symptoms indicative of neuroendocrine activity like palpitation, hypertension, or diaphoresis. Lab investigations were within normal limits. Chest X-ray showed a well-defined heterogeneous lesion in the left upper zone (Figure 1). Contrast-enhanced computed tomography (CECT) of the thorax showed a well-defined, smoothly marginated, heterogeneously enhancing soft tissue density lesion measuring ~4.1×3.7×3.8 cm in the left upper lobe (Figures 2-3). The patient underwent bronchoscopy with biopsy, and the sample was sent for histopathology. Post-procedure, she was stable. The biopsy report showed features suggestive of a neoplasm with clear cell morphology. Immunohistochemical staining was done, which showed positive staining for CD56, synaptophysin, chromogranin, and Ki-67 and negative staining for cytokeratin, PAX-8, CK7, and HMB45, suggestive of paraganglioma (Figures 4-7). Our patient was subsequently referred to the cardiothoracic surgical team for the evaluation of resection and to oncology for multidisciplinary care.

## Discussion

Paraganglioma is a rare type of neuroendocrine tumor that arises from paraganglionic cells of neural crest origin and most commonly affects individuals in middle age. Overall, paragangliomas constitute an uncommon clinical entity, with a reported incidence ranging from two to eight cases per million population per year [1]. Among these, primary pulmonary paragangliomas represent an exceedingly rare subset, accounting for approximately 0.2% of cases, thereby making their recognition and diagnosis particularly challenging in routine clinical practice.

These tumors may develop from sympathetic paraganglia located in the adrenal gland and along the abdominal para-aortic region or from parasympathetic paraganglia distributed in the head, neck, chest, and other anatomical sites where paraganglionic tissue is abundant, including the mediastinum and posterior peritoneum [2,4]. Nearly 40% of paraganglioma cases are reported to be associated with inherited genetic mutations, highlighting the significant role of hereditary factors in disease pathogenesis [4].

From an anatomical and functional standpoint, the diverse distribution of paraganglionic tissue throughout the body contributes to the wide variability in clinical presentation, radiological appearance, and biological behavior of paragangliomas. The pulmonary location is especially uncommon, as paraganglia are sparsely distributed within lung tissue, which may explain the extreme rarity of primary pulmonary involvement. Consequently, pulmonary paragangliomas are often not initially considered in the differential diagnosis of lung masses, particularly when they lack classical biochemical or clinical features associated with catecholamine secretion.

In general, primary pulmonary paragangliomas are typically non-functional and asymptomatic, with most cases being detected incidentally during radiological imaging performed for unrelated clinical indications rather than through symptoms attributable to catecholamine overproduction [5]. Although the majority of paragangliomas exhibit benign biological behavior, approximately 25% may demonstrate malignant potential. In this context, immunohistochemical analysis plays a crucial role in establishing the correct diagnosis and excluding histological mimics [6].

CT, while valuable for lesion detection and anatomical characterization, has limited specificity, as it is often difficult to reliably differentiate paragangliomas from other primary or metastatic pulmonary tumors based solely on imaging features [7]. As per the literature, patients with resectable primary pulmonary paraganglioma demonstrate excellent five-year survival (~90%), and outcomes are significantly poorer in unresectable or metastatic disease, with survival rates of only 40-50%.

In the present case, the patient presented with a pulmonary mass that was initially considered histopathologically suspicious for clear cell carcinoma based on routine microscopic evaluation. However, subsequent immunohistochemical analysis revealed positivity for markers characteristic of paraganglioma, thereby confirming the final diagnosis. Chromogranin A and synaptophysin, which are commonly employed neuroendocrine markers, demonstrated strong expression, with chromogranin A reflecting the presence of dense core secretory granules and synaptophysin being associated with synaptic vesicle membranes. Notably, both markers are known to remain expressed even in high-grade neuroendocrine tumors of the lung, thereby supporting their diagnostic utility in challenging cases [8].

However, in the present case, the patient exhibited no clinical, biochemical, or familial features suggestive of an underlying hereditary syndrome. Therefore, this case represents an exceptionally rare instance of a primary pulmonary paraganglioma occurring as a non-functional, sporadic variant. 

## Conclusions

Primary pulmonary paragangliomas are exceptionally uncommon neuroendocrine tumors, and their clinical behavior is not yet fully understood due to the limited number of reported cases. The absence of catecholamine-related symptoms often delays clinical suspicion, leading to frequent misclassification as other primary lung malignancies or metastatic lesions. Histologically, these tumors may demonstrate overlapping features with clear cell carcinoma or other neuroendocrine neoplasms, further complicating diagnosis. Immunohistochemistry thus plays a pivotal role in distinguishing paragangliomas from morphologically similar entities. This case report emphasizes the importance of considering rare diagnoses when evaluating patients with lung tumors and highlights the critical role of immunohistochemistry in achieving an accurate diagnosis. By identifying atypical markers, immunohistochemical testing can help distinguish rare tumors like pulmonary paraganglioma from more common malignancies, ensuring appropriate diagnosis and treatment.

However, given the potential for malignant transformation and late recurrence, long-term follow-up is strongly recommended. Genetic counseling and testing should be considered even in apparently sporadic cases, as occult germline mutations may still be present. Reporting such rare cases contributes valuable insights into the clinicopathological spectrum of pulmonary paragangliomas and aids in improving diagnostic accuracy and patient management strategies.

## References

[1] Fiorentino G, Annunziata A, De Rosa N (2015). Primary paraganglioma of the lung: a case report. J Med Case Rep.

[2] Tobón A, Velásquez M, Pérez B, Zúñiga V, Sua LF, Fernández-Trujillo L (2020). Pathologic features and clinical course of a non-functioning primary pulmonary paraganglioma: a case report. Ann Med Surg (Lond).

[3] De Palma A, Lorusso M, Di Gennaro F (2018). Pulmonary and mediastinal paragangliomas: rare endothoracic malignancies with challenging diagnosis and treatment. J Thorac Dis.

[4] Gimenez-Roqueplo AP, Robledo M, Dahia PL (2023). Update on the genetics of paragangliomas. Endocr Relat Cancer.

[5] Juhlin CC (2021). Challenges in paragangliomas and pheochromocytomas: from histology to molecular immunohistochemistry. Endocr Pathol.

[6] Huang X, Liang QL, Jiang L (2015). Primary pulmonary paraganglioma: a case report and review of literature. Medicine (Baltimore).

[7] Brink I, Hoegerle S, Klisch J, Bley TA (2005). Imaging of pheochromocytoma and paraganglioma. Fam Cancer.

[8] Pelosi G, Sonzogni A, Harari S (2017). Classification of pulmonary neuroendocrine tumors: new insights. Transl Lung Cancer Res.

